# Integrative Bioinformatics Analysis Incorporating Artificial Intelligence and Machine Learning Identifies Putative Pathogenic HNF1B Missense Variants Associated with HNF1B-MODY

**DOI:** 10.3390/ijms27208966

**Published:** 2026-10-09

**Authors:** Hitham Aldharee

**Affiliations:** 1 Department of Pathology, College of Medicine, Qassim University, Buraidah 52571, Saudi Arabia; h.aldharee@qu.edu.sa; 2 Department of Diabetes Center, Medical City, Qassim University, Buraidah 52571, Saudi Arabia

**Keywords:** HNF1B, artificial intelligence, MODY-5, diabetes mellitus, genetics

## Abstract

Maturity-onset diabetes of the young type 5 (MODY5 or *HNF1B*-MODY) is a rare form of diabetes caused by variants in *HNF1B*. Patients with MODY5 exhibit high blood sugar levels and symptoms commonly associated with both type 1 and type 2 diabetes (T1D, T2D), along with kidney and pancreatic issues. Nonetheless, diagnosis, treatment and management approaches depend on specific genotype–phenotype associations. Interpretation of *HNF1B* missense variants remains challenging because of conflicting classifications and limited clinical evidence. This study aimed to improve the interpretation of *HNF1B* missense variants with conflicting evidence regarding pathogenicity and thereby advance the genetic and molecular understanding of *HNF1B*-MODY. An integrative computational approach combining artificial intelligence (AI), machine learning (ML), and conventional bioinformatic tools, including Panther, SIFT, SNAP2, Meta-SNP, PhD-SNP, PolyPhen-2, and AlphaMissense, was used to identify deleterious missense variants in the *HNF1B* gene. The variants were further processed to determine their effects on protein conformation, stability, function, and conservation across species. Among 982 reported variants retrieved from the ClinVar database, 368 were classified as missense. Only 47 missense variants were labelled with conflicting classification and reported with clinical uncertainty. Consensus analysis by all tools identified five candidate variants (His153Arg, Asn298Asp, Arg303His, Lys305Glu, and Val458Gly), representing 10.6% (5/47) as potentially pathogenic. AlphaMissense classified all five as likely pathogenic, with scores ranging from 0.8921 to 1.000. Secondary structure analysis indicates that the His153Arg, Asn298Asp, Arg303His, and Lys305Glu variants are in the helical region, while the Val458Gly variant is located within the coil region. Interestingly, all five filtered variants are predicted by the I-Mutant and MuPro tools to reduce protein stability. The three-dimensional (3D) protein structures were constructed using the Deep Learning-Based Iterative Threading Assembly Refinement (D-I-TASSER) tool and visualised by UCSF Chimera. Structural modelling showed similarity between the mutant and wild-type models, with RMSD values ranging from 0.77 to 1.36 Å. The multiple sequence alignments show that amino acids at variant sites across all variants are highly conserved among species. These findings show that combining traditional bioinformatics with artificial intelligence and machine learning improves the interpretation of *HNF1B* missense variants with clinical uncertainty. Integrating computational predictions with structural, evolutionary, and clinical data provides a framework for prioritising variants for further functional and clinical investigation.

## 1. Introduction

Maturity-onset diabetes of the young, type 5 (MODY-5), is considered one of the rare forms of diabetes, typically affecting individuals under 25 years of age [1,2,3]. *Horikawa* first identified MODY-5, which has been linked to variants in a single gene called Hepatocyte Nuclear Factor 1-Beta (*HNF1B*; OMIM #189907) [4], which is involved in the development of the pancreas, kidneys, and other organs [5]. MODY-5 is rare, accounting for less than 5% of all MODY cases [1]. MODY-5 is mainly inherited in an autosomal dominant manner, meaning if one parent carries the defective gene, their child has a 50% chance of inheriting it [1,6].

The *HNF1B* gene, located on chromosome 17q12, comprises nine exons and encodes a 557-amino-acid protein. This protein, known as HNF1B, is a transcription factor that regulates genes crucial for normal organ development and blood sugar regulation [7]. Variations in this gene can lead to diabetes, as well as kidney problems such as cysts, abnormalities, or even complete kidney failure [1,5,7]. Some people may also experience issues with reproductive organs or liver enzyme levels [8]. Because of this, MODY-5 is not simply a form of diabetes, but a syndrome that affects multiple systems. While this study emphasizes *HNF1B*-associated diabetes, *HNF1B* variants are linked to a wider multisystem disease, especially involving renal and urogenital issues, and are not limited to the MODY phenotype.

The first pathogenic variant of the *HNF1B* gene was identified in a Japanese family with MODY in 1997 [9]. Disease- associated *HNF1B* variants include missense, nonsense, frameshift, and splice mutations. The variants have been classified according to American College of Medical Genetics (ACMG) guidelines as benign (B), likely benign (LB), pathogenic (P), likely pathogenic (LP), or of unknown significance (VUS [10]. Notably, several *HNF1B* missense variants found in individuals with hyperglycaemia are marked as ‘conflicting classifications’ in the ClinVar database, indicating ongoing debate among clinicians and researchers about whether these variants are harmful or simply common variations without medical significance. Given the limited number of cases, bioinformatic tools can assist in predicting how genetic variations affect protein stability, structure, and function, and ultimately lead to disease.

Previous studies have examined selected *HNF1B* variants associated with MODY [11,12]. However, some *HNF1B* missense variants with conflicting ClinVar classifications remain insufficiently characterised. The present study focuses on this group by integrating multiple computational prediction methods with analyses of protein stability, structural modelling, evolutionary conservation, and existing clinical data to identify variants that consistently show evidence of potential functional impact.

This study aims to improve understanding of the causes and mechanisms of *HNF1B*-MODY by investigating uncertain missense variants reported in individuals in the ClinVar database and assessing their potential impact on HNF1B protein function or expression using artificial intelligence (AI) and bioinformatic tools. The findings aim to support the diagnosis and management of individuals with MODY-5 and to refine management strategies for patients and their families.

## 2. Results

### 2.1. Variant Recruitment and Selection

In ClinVar, we identified approximately 982 germline variants in the *HNF1B* gene. Of these, 683 are single-nucleotide polymorphisms (SNPs), with the remainder distributed across other variant types. The SNPs predominantly resulted in 368 missense molecular consequences, with the remaining variants classified as nonsense, splice-site, UTR, or ncRNA. From this set, we focused on 47 missense variants that alter the amino acid sequence and are classified as having a “conflicting classification of pathogenicity” for further bioinformatic analysis.

### 2.2. Predicting the Pathogenicity of Missense Variants in HNF1B

A total of 47 missense variants were analysed using several bioinformatic tools: Meta-SNP, PolyPhen2, PANTHER, SIFT, PhD-SNP, and SNAP2 (Table S1). Variants with SIFT scores of 0.05 or lower were classified as deleterious, while those with PolyPhen2 scores above 0.85 were deemed probably damaging. The combined analysis identified five variants that were predicted damaging by all six tools (Table 1). However, individual tool results varied markedly. For example, PhD-SNP and SNAP2, both AI- and ML-based, identified 11 and 32 variants as disease-causing, respectively. Other tools, such as PANTHER, SIFT, and Meta-SNP, reported 19, 16, and 13 variants, respectively, as pathogenic. PolyPhen2 labelled 32 variants as probably damaging. Ultimately, only variants classified as pathogenic by all tools were selected for further study.

With lower agreement thresholds, five out of six tools predicted eight more variants as damaging (S465R, G285D, R261G, E260D, R235Q, R235W, P90T, G76C), while one variant (L12F) was predicted as damaging by four tools. Complete prediction results for all variants are available in Supplementary Table S2.

### 2.3. AlphaMissense Variants Pathogenicity Prediction

**Figure 1:**
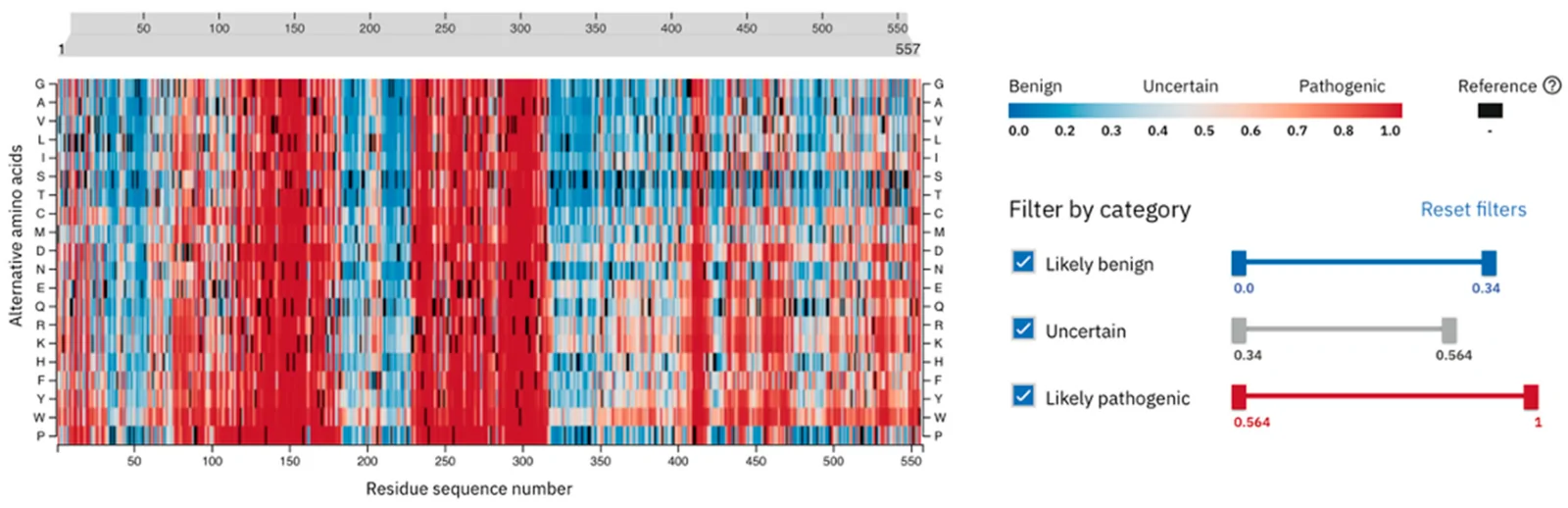
AlphaMissense heatmap analysis of *HNF1B* missense variants, showing the variant categorisation scoring system (Likely benign, Uncertain, Likely pathogenic).

We further analyse the five variants previously deemed pathogenic by all tools, using the AlphaMissense tool, a cutting-edge AI-based predictor. The results also suggest that all five are labelled as likely pathogenic (Table 2), with scores ranging from 0.89 to 1 (Figure 1).

### 2.4. Predicting Protein Stability

Further analysis of the protein stability of the filtered pathogenic variants was conducted using the MUpro and I-Mutant tools. The results indicate that all variants reduce protein stability (Table 3).

### 2.5. Predicting Protein Secondary Structure 

Using the Quick2D tool, we analyse the secondary structures of the mutated residues. The results indicate that His153Arg, Asn298Asp, Arg303His, and Lys305Glu are all located within the alpha-helical region, whereas Val458Gly is found in the coil region (Table 4).

### 2.6. Predicting Protein 3D Structure 

**Figure 2:**
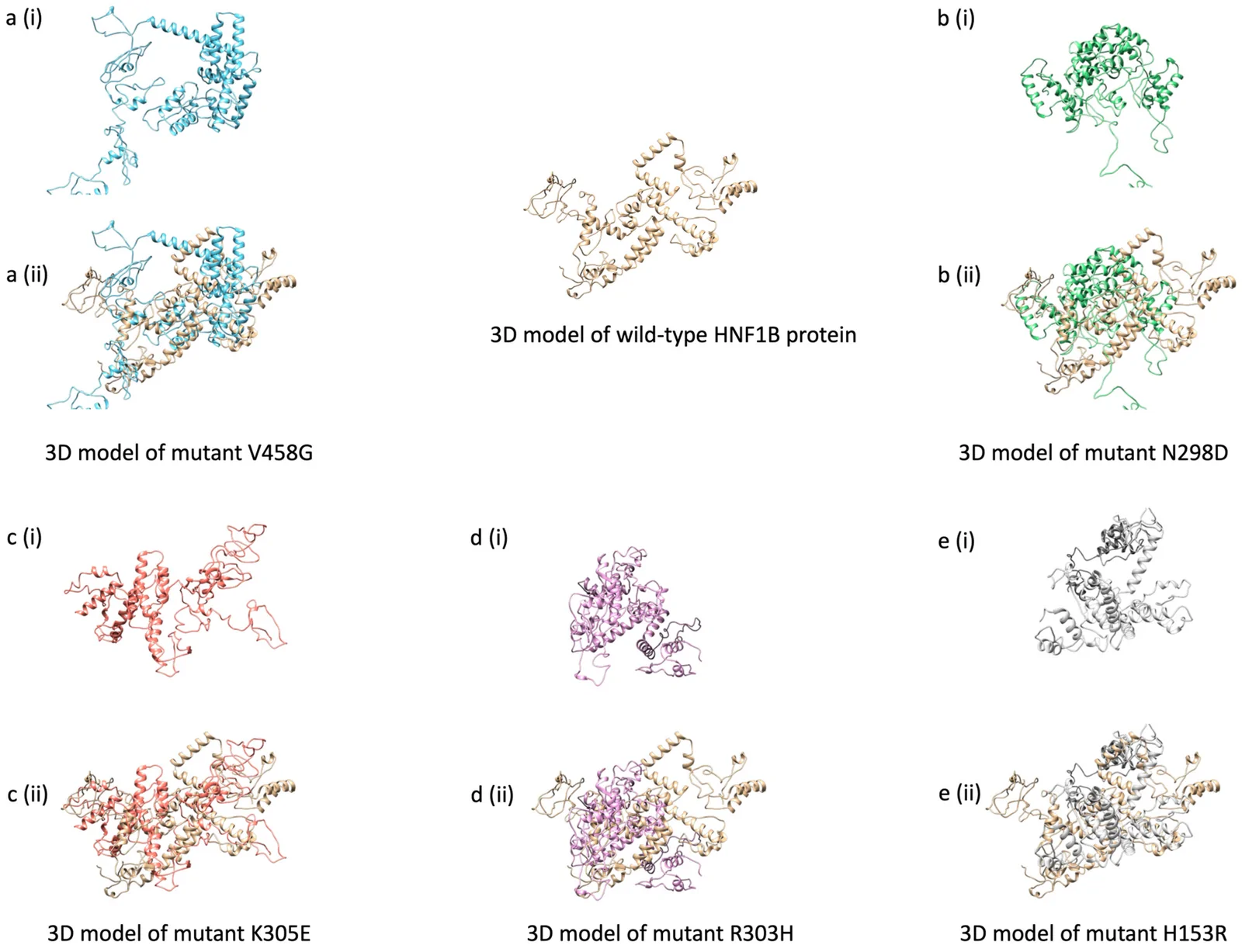
Three-dimensional structural analysis of HNF1B mutants includes: (**ai**,**bi**,**ci**,**di**,**ei**) predicted models of H153R, N298D, R303H, K305E, and V458G generated using D-I-TASSER; (**aii**,**bii**,**cii**,**dii**,**eii**) superimposed images of mutants and wild-type proteins highlighting localised conformational differences. All RMSD values ranged from 0.77 to 1.36 Å.

Structural modelling of the selected variants revealed notable similarity between the mutant and wild-type HNF1B models (Figure 2ai,bi,ci,di,ei). When superimposed, the models showed RMSD values of 0.77 to 1.36 Å, indicating that the overall predicted protein structures were largely conserved (Figure 2aii,bii,cii,dii,eii). Given the small deviations and the inherent variability of computational modelling, it is not possible to confidently attribute the structural differences to specific amino acid substitutions.

### 2.7. Conservation of HNF1B Amino Acids Across Various Species

**Figure 3:**
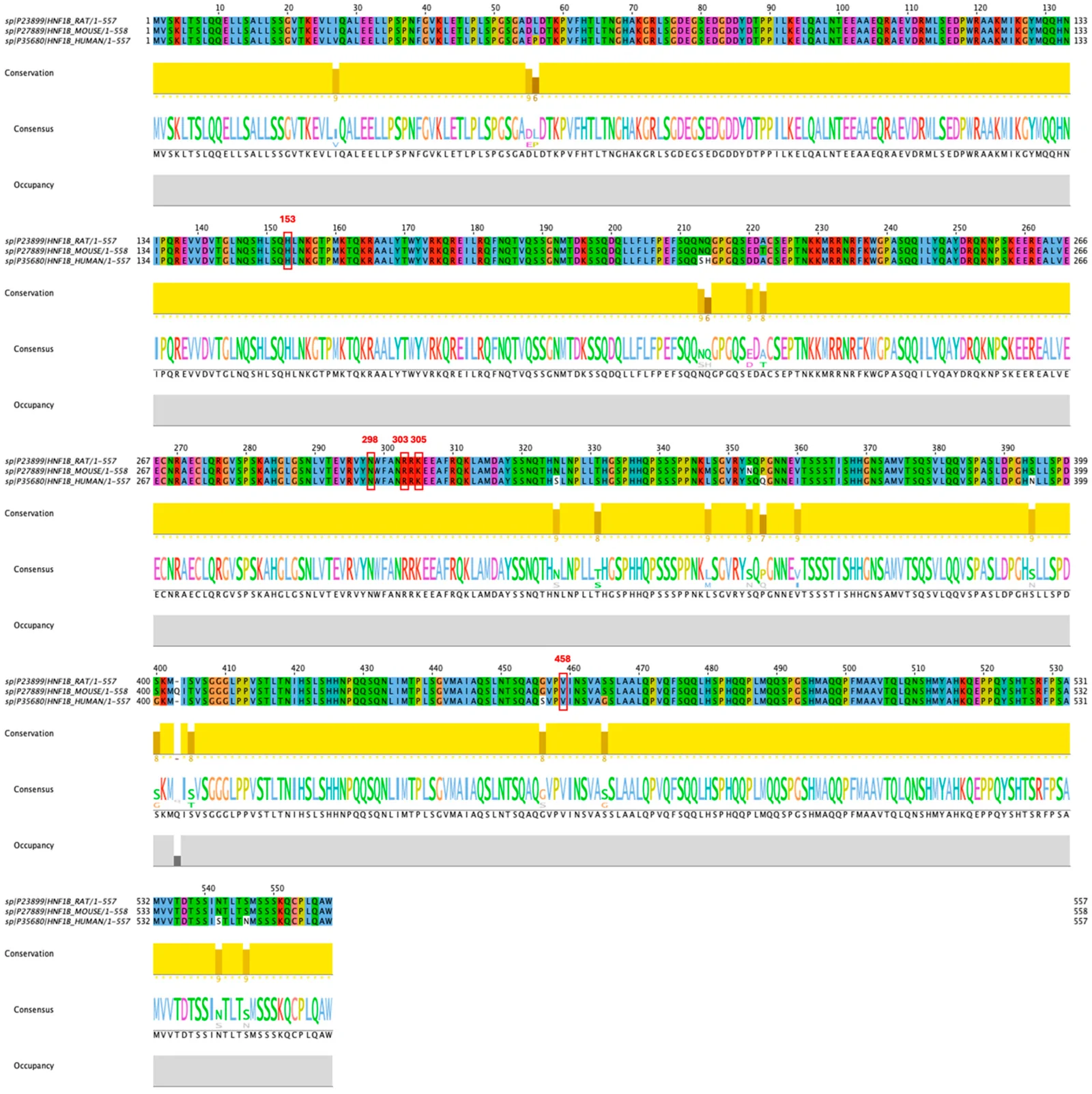
Conservation analysis of amino acids in the HNF1B protein using Clustal Omega (Version 1.2.4). The variants we focus on are highlighted with red boxes.

The multiple sequence alignment in Jalview shows that the amino acids at the sites of change across all variants are highly conserved across human, rat, and mouse species (Figure 3). This indicates that these residues are essential for function or have been preserved through evolution, making alterations at these positions potentially significant.

## 3. Discussion 

Bioinformatic analysis is now a widely used method for determining the effects of genetic variants, supporting diagnosis and disease treatment, and enabling cost and time savings before and after laboratory tests. Here, 47 *HNF1B* missense variants reported with conflicting classifications were comprehensively assessed using an integrated bioinformatics approach that combines AI and ML with conventional bioinformatics tools.

Despite variability among individual prediction tools, consensus from multiple computational methods indicates that His153Arg, Asn298Asp, Arg303His, Lys305Glu, and Val458Gly are likely pathogenic variants associated with *HNF1B*-MODY. Evidence from protein stability studies, structural modelling, evolutionary conservation, and clinical reports further supports their potential functional significance. MUpro and I-Mutant both predict that all five amino acid changes decrease HNF1B protein stability, potentially disrupting folding, altering conformational dynamics, and impairing transcription-factor activity.

Domain annotation and available structural data suggest that His153 is part of the HNF1B DNA-binding region, while Asn298, Arg303, and Lys305 reside within the homeodomain. The experimentally determined HNF1B DNA-binding structure (PDB: 2H8R) covers residues 91–310, and the isolated homeodomain structure (PDB: 2DA6) includes residues 233–321. Secondary-structure prediction shows these four residues are within predicted α-helical regions; however, their presence in these regions does not necessarily indicate a disruption of α-helical structure [13]. These substitutions may modify the structure, charge distribution, or stability of the DNA-binding interface, potentially reducing affinity or specificity. Conversely, Val458Gly is situated in a coil region of the C-terminal transactivation domain, which helps recruit transcriptional regulators and activate HNF1B target genes; thus, Val458Gly may impair transcriptional activation without directly affecting DNA binding [13]. Overall, the localisation of these variants across the DNA-binding and activation domains suggests they may influence HNF1B function through different but complementary molecular mechanisms. Prior experimental data indicate that Arg303His may have a functional role. Mirshahi et al. investigated the HNF1B p.Arg303His variant using luciferase reporter assays and observed a mild decrease in transactivation of HNF1B target promoters, including approximately an 11% reduction in PKHD1 promoter activity. This variant was also identified in individuals with renal phenotypes. These results support the predicted functional significance of Arg303His.

Structural modelling aligns with the predicted functional importance of the selected variants. Comparison of the wild-type and mutant HNF1B protein structures showed RMSD values of 0.77–1.36 Å, indicating that the overall tertiary structure remains largely intact despite amino acid changes. Because these deviations are small and computational models inherently fluctuate, the observed structural differences cannot be confidently attributed to specific amino acid substitutions. Separately, both MUpro and I-Mutant independently predicted decreased protein stability for all five variants, though the confidence and degree of these predictions differed among them. This indicates a possible impact on HNF1B stability but should not be viewed as definitive quantitative proof of protein destabilization.

His153, Asn298, Arg303, Lys305, and Val458 were conserved across the three mammalian species analyzed (human, mouse, and rat), offering qualitative evidence of conservation at these sites. However, because of the limited phylogenetic diversity of these species, this finding was not regarded as independent evidence of pathogenicity.

Finally, published clinical evidence for the five *HNF1B* variants is summarised in Table 5. Only Val458Gly and Lys305Glu have been reported in individuals with clinical features consistent with *HNF1B*-related disease. For Val458Gly, published clinical evidence provides additional context for the computational findings. The variant has been reported in individuals with *HNF1B*-related MODY, including a 45-year-old patient with diabetes, preserved C-peptide (0.8 nmol/L), normal BMI (23.4 kg/m^2^), negative islet autoantibodies, hyperlipidaemia, and a family history of diabetes and kidney disease. However, no published clinical evidence was found for His153Arg, Asn298Asp, or Arg303His. This limited clinical data underscores a significant challenge in interpreting *HNF1B* variants, as the absence of a clear genotype–phenotype correlation often leads to uncertain or conflicting clinical classifications. In such cases, integrative bioinformatics approaches can help interpret variants by combining computational predictions with structural, evolutionary, and clinical data.

### Limitations

Since many prediction tools rely on similar methods or input features, their concordance should not be regarded as multiple independent pieces of evidence for pathogenicity. In addition, the consensus-based filtering strategy was not evaluated against an independent set of known pathogenic and benign *HNF1B* variants, and its performance metrics like sensitivity, specificity, AUC, and MCC were not determined. Consequently, this method should be regarded as a computational filtering approach rather than a thoroughly validated classification model. The stability analysis was restricted to sequence-based predictions and did not involve quantitative structure-based ΔΔG calculations. As a result, these findings should be viewed as predictions rather than direct experimental evidence of protein destabilization.

While computational methods cannot replace experimental validation or thorough clinical investigation, they provide a systematic way to identify high-priority variants that require further functional and clinical assessment. The computational workflow in this study could also be adapted into a semi-automated pipeline, making it easier to apply to other genes and variant groups with similar interpretation challenges.

## 4. Material and Methods

### 4.1. Variants Recruitment and Selection

A total of 982 variants in the *HNF1B* gene were retrieved from the ClinVar database (https://www.ncbi.nlm.nih.gov/clinvar/, accessed on 12 March 2025). ClinVar was selected because this study specifically focuses on variants with conflicting clinical classifications, which are explicitly reported in this database. Of the 982 *HNF1B* variants retrieved, only 368 missense variants in the *HNF1B* gene were identified. The dataset was manually filtered using Microsoft Excel, and 47 missense variants labelled with conflicting classifications were selected for further analysis. Variant nomenclature was standardised using *HNF1B* reference transcript NM_000458IN in accordance with the Human Genome Variation Society (HGVS) guidelines. The selected variants were subsequently analysed using multiple bioinformatic tools. The human HNF1B protein sequence from UniProt ID: P35680 was utilized for protein-level analysis.

### 4.2. Predicting Pathogenicity of Variants 

The selected variants were analysed with the following bioinformatics tools to assess their potential to cause disease:

PHD-SNP (https://snps.biofold.org/phd-snp/phd-snp.html, accessed on 20 March 2025) is a machine learning-based tool built on a support vector machine (SVM). The tool is trained to identify disease-associated nsSNPs using only sequence information. Variants are classified as either disease-causing or neutral.

SNAP-2 (https://bio.tools/snap2, accessed on 20 March 2025) is a machine learning-based tool trained using neural network methods (NNMs). It predicts how variants affect proteins by analysing various sequence properties and indicates whether the variant is damaging or neutral.

PANTHER (http://pantherdb.org/, accessed on 25 March 2025) uses evolutionary information to predict the functional effects of amino acid substitutions. PANTHER scores were interpreted using a threshold of 0.5, with scores > 0.5 indicating a predicted disease-associated effect.

SIFT (http://sift.bii.a-star.edu.sg/, accessed on 25 March 2025) predicts whether an amino acid change damages a protein. The result appears as “damage (deleterious)” or “benign (tolerant)” depending on a cut-off value. Scores range from 0 to 1, with scores ≤ 0.05 classified as deleterious and scores > 0.05 classified as tolerated.

Meta-SNP (https://snps.biofold.org/meta-snp/, accessed on 25 March 2025) is a random forest-based meta-predictor that integrates predictions to classify missense variants as disease-associated or neutral. Meta-SNP generates a probability score ranging from 0 to 1, with scores > 0.5 classified as disease-associated and scores ≤ 0.5 classified as neutral.

Polyphen2 (http://genetics.bwh.harvard.edu/pph2/, accessed on 28 March 2025) predicts the potential impact of amino acid substitutions on protein structure and function using sequence- and structure-based features. The prediction classifies the effect as probably damaging, possibly damaging, or benign. The classification depends on the position-specific independent count (PSIC) score, which ranges from 0 to 1, with values near 0 indicating a benign change and those near 1 suggesting it is probably damaging.

### 4.3. AlphaMissense Variants Pathogenicity Prediction 

AlphaMissense (https://www.science.org/doi/10.1126/science.adg7492, accessed on 25 March 2025) is an advanced tool that forecasts the effects of missense variants on proteins. In this research, precomputed AlphaMissense predictions from the online resource were utilized. The tool employs a deep learning model to assign a pathogenicity score between 0 and 1 for each amino acid change, where higher scores suggest greater potential for pathogenicity. Scores under 0.34 are considered likely benign, those between 0.34 and 0.564 are ambiguous, and scores above 0.564 are deemed likely pathogenic.

### 4.4. Predicting Protein Secondary Structure

The secondary structures of the normal HNF1B protein and the mutant forms, which were identified as pathogenic by all bioinformatic tools, were predicted using the Quick2D server (https://toolkit.tuebingen.mpg.de/tools/quick2d, accessed on 10 April 2025) [16]. The platform combines multiple algorithms, such as PSIPRED, to predict beta-sheet, alpha-helical, and coil regions.

### 4.5. Predicting Protein Stability 

The effects of amino acid substitutions on protein stability were analysed using MUpro (http://mupro.proteomics.ics.uci.edu, accessed on 15 April 2025) and I-Mutant 2.0 (https://folding.biofold.org/i-mutant/i-mutant2.0.html, accessed on 15 April 2025). The sequence-based predictions used the HNF1B amino acid sequence as an input. MUpro uses machine learning methods to predict whether a substitution increases or decreases stability, whereas I-Mutant 2.0 uses a support vector machine (SVM) to estimate stability changes following a single amino acid substitution.

### 4.6. Predicting Protein 3D Structure

Modelling of the 3D structure of the HNF1B wild-type protein and the selected variants predicted to be pathogenic by all bioinformatic tools was performed using the Deep Learning-Based Iterative Threading Assembly Refinement (D-I-TASSER) server (https://zhanggroup.org/D-I-TASSER/, accessed on 15 May 2025) [17]. This tool constructs a 3D protein structure by combining multiple sequence alignment (MSA) and deep learning to generate restraints, which are then integrated with I-TASSER fragment assembly simulations. This approach offers higher accuracy, particularly for large multidomain proteins, by applying domain splitting and reassembly steps. UCSF Chimera software (https://www.cgl.ucsf.edu/chimera/, accessed on 15 May 2025) was used for visualisation, enabling overlay of normal and mutant proteins for comparison. The wild-type and mutant models were overlaid in UCSF Chimera using their complete predicted protein structures, and RMSD values were derived from the structural alignment.

### 4.7. Conservation Analysis 

**Figure 4:**
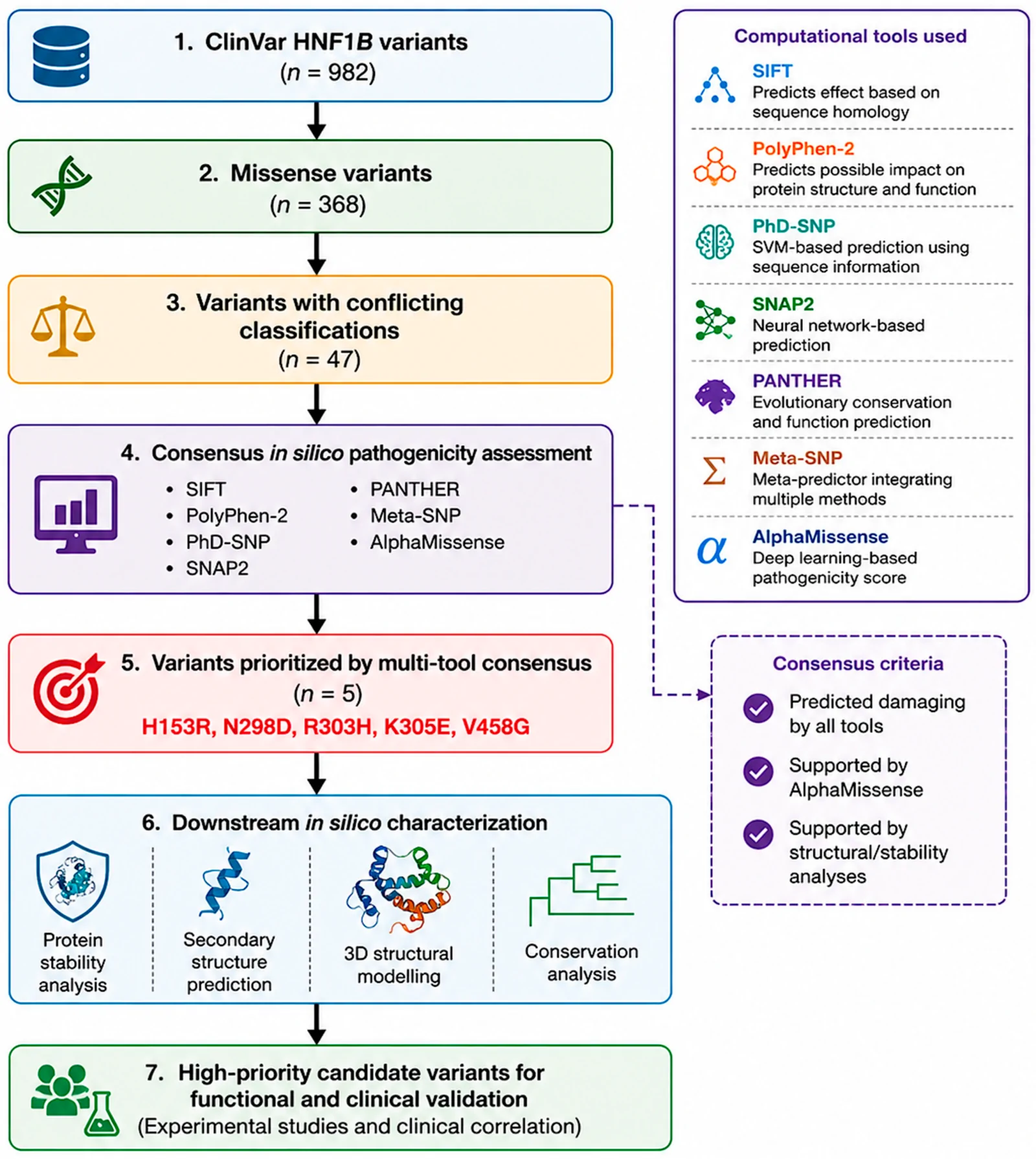
Workflow outlining the variant filtering process and the consensus computational approach used to determine variant pathogenicity in this study. Of 982 *HNF1B* variants in ClinVar, 47 missense variants with conflicting classifications were selected for further computational consensus analysis. Of these, five high-priority candidate variants were identified for detailed in silico structural and functional characterisation.

The conservation of the HNF1B protein across species was assessed using Jalview software (v2.11.4.1) [18]. Amino acid sequences from human, rat, and mouse were obtained from the NCBI HomoloGene database (https://www.ncbi.nlm.nih.gov, accessed on 19 May 2025). In Jalview, a multiple sequence alignment (MSA) and consensus analysis were performed, highlighting conserved amino acids across species. Figure 4 summarises the overall workflow for variant filtering and analyses. All computational analyses were conducted with the web-based tools and software mentioned above.

## 5. Conclusions

This study identified five *HNF1B* missense variants (His153Arg, Asn298Asp, Arg303His, Lys305Glu, and Val458Gly) that consistently show predicted damaging effects despite conflicting ClinVar classifications. All five variants affect highly conserved residues, and MUpro and I-Mutant predict decreased protein stability. Structural modelling indicates that the overall protein structure remains intact, with RMSD values of 0.77–1.36 Å. Future research may incorporate luciferase reporter assays to quantify transcriptional activity, electrophoretic mobility shift assays (EMSAs) to assess DNA-binding affinity, and studies of protein stability and degradation to investigate the structural effects of the identified substitutions. Additionally, family segregation analyses, prospective studies of individuals suspected of having *HNF1B*-related disease, and advanced functional genomic techniques such as deep mutational scanning could help refine genotype–phenotype associations and improve variant classification.

## Figures and Tables

**Table 1:** Variants are commonly predicted to be pathogenic by all bioinformatics prediction tools.

	Variant	dbSNP ID	Protein Change	Panther		PhD-SNP		SIFT		SNAP2		Meta-SNP		PolyPhen-2	
				Score	Pred	Score	Pred	Score	Pred	Score	Pred	Score	Pred	Score	Pred
1	NM_000458.4 (*HNF1B*): c.1373T>G (p.Val458Gly)	rs375644184	V458G	0.584	D	0.610	D	0.010	D	0.660	D	0.644	D	0.991	PD
2	NM_000458.4 (*HNF1B*): c.913A>G (p.Lys305Glu)	rs2147545316	K305E	0.683	D	0.766	D	0.010	D	0.730	D	0.720	D	0.999	PD
3	NM_000458.4 (*HNF1B*): c.908G>A (p.Arg303His)	rs2033692404	R303H	0.645	D	0.789	D	0.020	D	0.800	D	0.779	D	0.998	PD
4	NM_000458.4 (*HNF1B*): c.892A>G (p.Asn298Asp)	rs1598841002	N298D	0.644	D	0.742	D	0.030	D	0.735	D	0.719	D	0.972	PD
5	NM_000458.4 (*HNF1B*): c.458A>G (p.His153Arg)	rs1598848762	H153R	0.784	D	0.701	D	0.010	D	0.750	D	0.605	D	1.00	PD

Key: dbSNP: Single Nucleotide Polymorphism Database, Pred: Prediction, D: Disease, PD: Probably Damaging.

**Table 2:** Prediction of alpha-missense for the pathogenic missense variants reported in Table 1.

	dbSNP ID	Protein Change	Alpha-Missense	
			Score	Prediction
1	rs375644184	V458G	0.8921	likely pathogenic
2	rs2147545316	K305E	0.9999	likely pathogenic
3	rs2033692404	R303H	0.9927	likely pathogenic
4	rs1598841002	N298D	1.0	likely pathogenic
5	rs1598848762	H153R	0.9998	likely pathogenic

**Table 3:** Predicted effects of the five selected *HNF1B* variants on protein stability.

	dbSNP ID	Protein Changes	I-Mutant		MuPro	
			Stability	RI	Stability	Score
1	rs375644184	V458G	Decrease	9	Decrease	−2.34
2	rs2147545316	K305E	Decrease	6	Decrease	−0.11
3	rs2033692404	R303H	Decrease	9	Decrease	−1.35
4	rs1598841002	N298D	Decrease	4	Decrease	−1.22
5	rs1598848762	H153R	Decrease	5	Decrease	−0.52

**Table 4:** Secondary structure and evolutionary conservation of the selected *HNF1B* variants.

Variant	Residue Position	Secondary Structure	Evolutionary Conservation
V458G	458	Coil	Highly conserved
K305E	305	Helix	Highly conserved
R303H	303	Helix	Highly conserved
N298D	298	Helix	Highly conserved
H153R	153	Helix	Highly conserved

**Table 5:** Current published clinical reports of the identified *HNF1B* missense variants.

	Variant	Protein Change	Published Clinical Evidence	Key Clinical Features	Reference
1	NM_000458.4 (*HNF1B*): c.1373T>G (p.Val458Gly)	V458G	Reported in a proband with early- diabetes and a positive family history of diabetes.	Age at diagnosis: 45, BMI at diagnosis: 23.4 kg/m^2^, C-peptide at diagnosis: 0.8 nmol/L, A1C at diagnosis: 6.4%, Hyperlipidaemia.	[14]
2	NM_000458.4 (*HNF1B*): c.913A>G (p.Lys305Glu)	K305E	Identified in a patient with suspected MODY; detailed individual clinical characteristics were not reported.	Not Reported	[15]
3	NM_000458.4 (*HNF1B*): c.908G>A (p.Arg303His)	R303H	No published clinical report identified	Not Available	___
4	NM_000458.4 (*HNF1B*): c.892A>G (p.Asn298Asp)	N298D	No published clinical report identified	Not Available	___
5	NM_000458.4 (*HNF1B*): c.458A>G (p.His153Arg)	H153R	No published clinical report identified	Not Available	___

## Data Availability

The original contributions presented in this study are included in the article/Supplementary Materials. Further inquiries can be directed to the corresponding author.

## Supplementary Materials

The following supporting information can be downloaded at: https://www.mdpi.com/article/10.3390/ijms27208966/s1.
