# Supplementary material for: Integrative Bioinformatics Analysis Incorporating Artificial Intelligence and Machine Learning Identifies Putative Pathogenic HNF1B Missense Variants Associated with HNF1B-MODY
Source: Int J Mol Sci. 2026 Oct 9;27(20):8966. doi: 10.3390/ijms27208966 (PMC13705104; doi:10.3390/ijms27208966)
Supplement: Supplementary file 1 [file ijms-27-08966-s001.zip › ijms-4520105-supplementary.pdf]

Supplementary Material (S1)

Table S1: ClinVar classification and submission characteristics of the five prioritised HNF1B missense variants.

Variant	ClinVar classification	Nature of conflicting classifications	No. of submissions	Review status
V458G	Conflicting classifications	Uncertain significance; Benign ; Likely benign	5	Criteria provided, conflicting classifications
K305E	Conflicting classifications	Uncertain significance; Pathogenic	3	Criteria provided, conflicting classifications
R303H	Conflicting classifications	Pathogenic ; Likely pathogenic ; Likely risk allele ; Uncertain significance	4	Criteria provided, conflicting classifications
N298D	Conflicting classifications	Uncertain significance; Pathogenic	3	Criteria provided, conflicting classifications
H153R	Conflicting classifications	Likely risk allele ; Uncertain significance	3	Criteria provided, conflicting classifications

Table S2:

Chr:bp	Alleles	AA	AA cord	Protein Change	Panther		PhD-SNP		SIFT		SNAP		Meta-SNP		PolyPhen-2	
					Score	Pred	Score	Pred	Score	Pred	Score	Pred	Score	Pred	Score	Pred
37699092	T>C	Met/Thr	546	M546T	0.418	N	0.163	N	0.050	D	0.640	D	0.149	N	0.891	PD
37699135	A>G	Met/Val	532	M532V,	0.237	N	0.387	N	0.180	N	0.555	D	0.213	N	0.985	PD
37701033	T>A	Met/Lys	495	M495K	0.471	N	0.471	N	0.210	N	0.570	D	0.449	N	0.961	PD
37701099	A>G	Gln/Arg	473	Q473R,	0.357	N	0.198	N	0.400	N	0.500	N	0.262	N	0.012	B
37701103	G>A	Val/Ile	472	V472I	0.172	N	0.028	N	0.440	N	0.300	N	0.083	N	0.133	B
37701121	C>G	Leu/Val	466	L466V	0.311	N	0.355	N	0.010	D	0.630	D	0.328	N	0.998	PD
37701122	C>G	Ser/Arg	465	S465R	0.493	N	0.587	D	0.030	D	0.790	D	0.730	D	1.00	PD
37701144	T>G	Val/Gly	458	V458G	0.584	D	0.610	D	0.010	D	0.660	D	0.644	D	0.991	PD
37704920	C>G	Gln/Glu	446	Q446E	0.347	N	0.064	N	0.250	N	0.520	D	0.084	N	0.994	PD
37705013	A>T	Thr/Ser	415	T415S	0.290	N	0.402	N	0.180	N	0.400	N	0.353	N	0.999	PD
37710601	G>A	Gly/Ser	370	G370S	0.122	N	0.031	N	0.530	N	0.520	D	0.081	N	0.234	B
37731615	C>T	Ser/Phe	342	S342F	0.678	D	0.107	N	0.600	N	0.270	N	0.130	N	0.012	B
37731616	T>C	Ser/Pro	342	S342P	0.572	D	0.154	N	0.460	N	0.270	N	0.130	N	0.989	PD
37731634	C>G	His/Asp	336	H336D	0.485	N	0.101	N	0.640	N	0.550	N	0.098	N	0.425	B
37731634	C>T	His/Tyr	336	H336Y	0.476	N	0.076	N	0.520	N	0.510	N	0.098	N	0.917	PD
37731657	C>T	Pro/Leu	328	P328L	0.200	N	0.053	N	0.680	N	0.305	N	0.069	N	0.000	B
37731666	G>A	Ser/Asn	325	S325N	0.068	N	0.123	N	1.000	N	0.225	N	0.110	N	0.000	B
37731678	A>G	Asn/Ser	321	N321S	0.141	N	0.058	N	0.480	N	0.305	N	0.071	N	0.000	B
37731691	G>T	Ala/Ser	317	A317S	0.301	N	0.218	N	0.670	N	0.205	N	0.253	N	0.916	PD
37731727	A>G	Lys/Glu	305	K305E	0.683	D	0.766	D	0.010	D	0.730	D	0.720	D	0.999	PD
37731732	G>A	Arg/His	303	R303H	0.645	D	0.789	D	0.020	D	0.800	D	0.779	D	0.998	PD
37731748	A>G	Asn/Asp	298	N298D	0.644	D	0.742	D	0.030	D	0.735	D	0.719	D	0.972	PD
37731786	G>A	Gly/Asp	285	G285D	0.859	D	0.758	D	0.731	N	0.785	D	0.765	D	1.00	PD
37733585	A>G	Arg/Gly	261	R261G	0.804	D	0.555	D	0.020	D	0.760	D	0.340	N	1.00	PD
37733586	G>C	Glu/Asp	260	E260D	0.572	D	0.352	N	0.040	D	0.575	D	0.514	D	0.745	PD

37733611	G>A	Arg/Gln	252	R252Q	0.737	D	0.265	N	0.240	N	0.530	D	0.340	N	1.00	PD
37733662	G>A	Arg/Gln	235	R235Q	0.737	D	0.536	D	0.140	N	0.735	D	0.647	D	1.00	PD
37733663	C>T	Arg/Trp	235	R235W	0.952	D	0.542	D	0.130	N	0.830	D	0.750	D	1.00	PD
37733704	A>T	Asp/Val	221	D221V	0.652	D	0.166	N	0.290	N	0.310	N	0.130	N	0.90	B
37739442	G>A	Arg/Gln	181	R181Q	0.246	N	0.112	N	0.640	N	0.385	N	0.101	N	1.00	PD
37739467	G>A	Val/Ile	173	V173I	0.197	N	0.146	N	0.240	N	0.270	N	0.137	N	0.731	PD
37739526	A>G	His/Arg	153	H153R	0.784	D	0.391	D	0.010	D	0.750	D	0.605	D	1.00	PD
37744541	G>A	Ser/Asn	115	S115N	0.369	N	0.193	N	0.070	N	0.375	N	0.254	N	0.117	B
37744571	A>G	Glu/Gly	105	E105G	0.334	N	0.270	N	0.020	D	0.570	D	0.313	N	1.00	PD
37744572	G>A	Glu/Lys	105	E105K	0.626	N	0.183	N	0.270	N	0.345	N	0.147	N	0.996	PD
37744617	C>A	Pro/Thr	90	P90T	0.839	D	0.445	N	0.040	D	0.625	D	0.551	D	0.942	PD
37744641	G>A	Asp/Asn	82	D82N	0.425	N	0.426	N	0.340	N	0.340	N	0.368	N	0.581	PD
37744654	C>G	Asp/Glu	77	D77E	0.308	N	0.335	N	0.440	N	0.370	N	0.337	N	0.999	PD
37744659	G>T	Gly/Cys	76	G76C	0.796	D	0.623	D	0.101	D	0.640	D	0.217	N	1.00	PD
37744703	T>G	Val/Gly	61	V61G	0.584	D	0.521	D	0.270	N	0.545	D	0.415	N	0.000	B
37744778	C>T	Ser/Phe	36	S36F	0.639	D	0.257	N	0.460	N	0.540	D	0.144	N	0.484	PD
37744812	G>T	Val/Leu	25	V25L	0.19	N	0.105	N	0.340	N	0.215	N	0.090	N	0.505	PD
37744851	C>T	Leu/Phe	12	L12F	0.66	D	0.21	N	0.020	D	0.675	D	0.388	N	1.00	PD
